# AGE-ASSOCIATED GRANZYME K-EXPRESSING CD8+ T-CELLS ENHANCE ATHEROSCLEROSIS IN MICE

**DOI:** 10.1093/geroni/igac059.784

**Published:** 2022-12-20

**Authors:** Daniel Tyrrell, Daniel Goldstein

**Affiliations:** University of Alabama at Birmingham, Birmingham, Alabama, United States; University of Michigan, Ann Arbor, Michigan, United States

## Abstract

A novel population of age-associated Granzyme K (GZMK)-expressing CD8 T cells was found in mice and humans. These cells enhance local tissue senescence and inflammation and are distinct from central memory and conventional Granzyme B- or Interferon γ-producing effector memory CD8 T cells. It is unknown whether these cells drive chronic disease; thus, we induced atherosclerosis in young (3-mo) and aged (18-mo) wild-type mice via the PCSK9-AAV model and used scRNAseq to demonstrate that this GZMK-CD8 T cell population homes to atherosclerotic lesions exclusively in aged mice. Neutralizing CD8 T cells demonstrates that GZMK-CD8 cells drive age-enhance atherosclerosis. Finally, we transferred GZMK-CD8 cells from different aged donors into young CD8-/- hosts and demonstrate that GZMK-CD8 cells from aged but not young donors drive atherosclerosis. In conclusion, we identified a novel role for this recently described population of aging-specific GZMK-expressing CD8+ T cells as a critical driver of chronic disease.

